# Hemoptysis Associated With Rasmussen Aneurysm

**DOI:** 10.7759/cureus.39006

**Published:** 2023-05-14

**Authors:** Sarah Ream, Abhay Raina, Juan B Figueroa-Casas

**Affiliations:** 1 Internal Medicine, Texas Tech University Health Sciences Center El Paso Paul L. Foster School of Medicine, El Paso, USA; 2 Radiology, Texas Tech University Health Sciences Center El Paso Paul L. Foster School of Medicine, El Paso, USA; 3 Pulmonary and Critical Care Medicine, Texas Tech University Health Sciences Center El Paso Paul L. Foster School of Medicine, El Paso, USA

**Keywords:** coil embolization, pseudoaneurysm, tuberculosis, rasmussen aneurysm, hemoptysis

## Abstract

Hemoptysis involves compromised pulmonary vasculature of bronchial versus pulmonary arterial origins, with both life-threatening and non-life-threatening causes. Life-threatening hemoptysis is uncommon. To date, published cases of Rasmussen aneurysm remain low and subsequently underrecognized. We report on a 63-year-old male from Mexico with a more than 30-pack-year smoking history, but no history of lung disease, who presented to the emergency department with cough and hemoptysis for one week. A computed tomography angiography (CTA) chest demonstrated a pseudoaneurysm and hemorrhage, consistent with a Rasmussen aneurysm. Interventional radiology performed a pulmonary angiography, and subsequent coil embolization of the tertiary feeding arteries was performed. This case demonstrates a rare pulmonary artery pseudoaneurysm, known simply as Rasmussen aneurysm, that was successfully managed with coil embolization and highlights the importance of considering the disease in the differential diagnosis for patients with hemoptysis.

## Introduction

Hemoptysis involves compromised pulmonary vasculature of bronchial versus pulmonary arterial origins, with both life-threatening and non-life-threatening causes. Life-threatening hemoptysis is uncommon. Since 1868, Rasmussen aneurysm, a pseudoaneurysm arising from the pulmonary artery, has been described relatively few times in the literature, with an estimated prevalence of 4%-8%, most often seen in regions of high tuberculosis (TB) burden [1-6]. To date, published cases of Rasmussen aneurysm remain low and subsequently underrecognized.

## Case presentation

**Figure 1:**
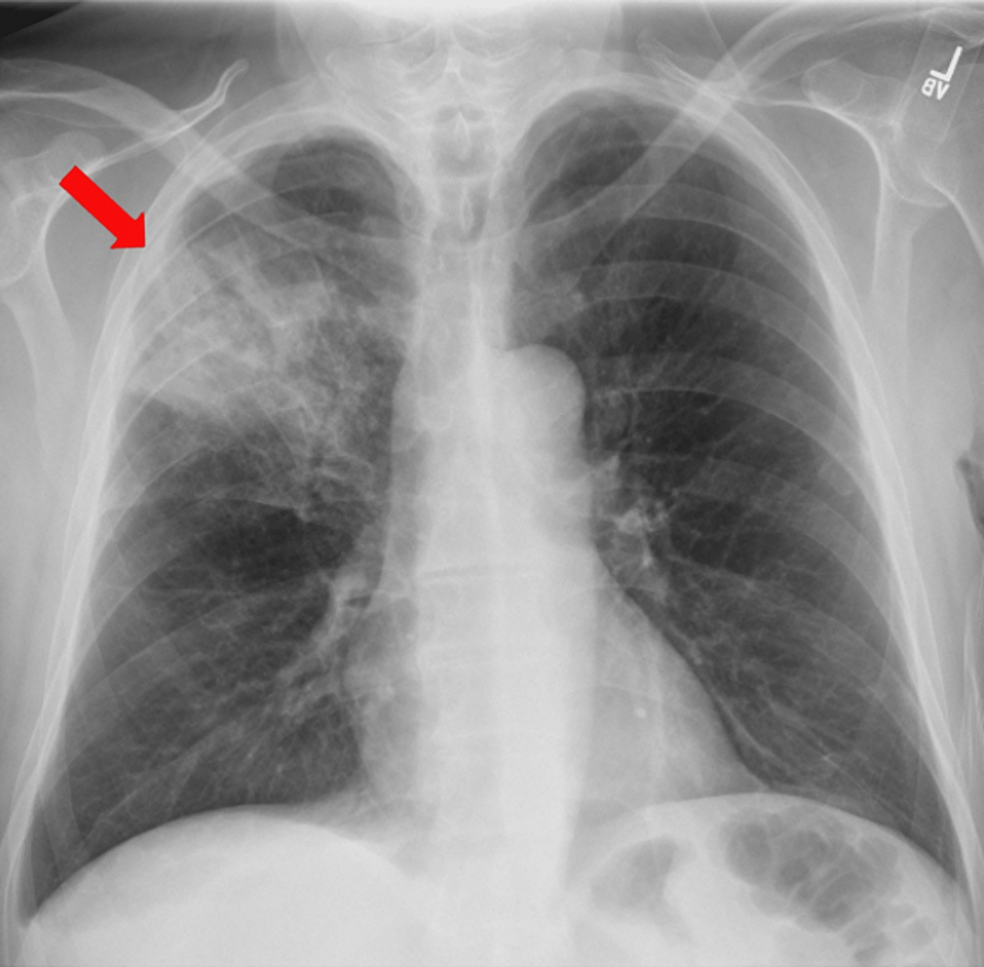
Chest radiograph from the day of admission demonstrating a right upper lobe cavitary lesion (red arrow).

We report on a 63-year-old male from Mexico with a more than 30-pack-year smoking history, but no history of lung disease, who presented to the emergency department with cough and an episode of large volume hemoptysis, estimated 200 mL, followed by daily mild to moderate hemoptysis approximately 10 to 50 mL for one week. The patient reported fevers and unintentional weight loss with new, acute onset of chills and night sweats. He appeared pale and cachectic with lungs clear bilaterally to auscultation. Initial laboratory results demonstrated a leukocytosis of 28.19 x 10^3^/mL, normocytic anemia with a hemoglobin of 10.5 G/DL, and platelet count of 323 x 10^3^/UL. A chest radiograph showed a large cavitary lesion involving the right upper lobe (Figure 1).

**Figure 2:**
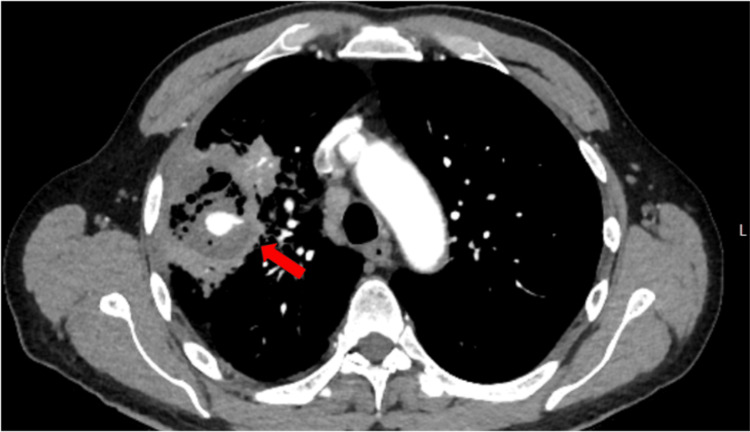
CTA chest axial view demonstrating an irregular right upper lobe complex cavitary lesion with internal contrast extravasation compatible with a pulmonary artery pseudoaneurysm and hemorrhage, consistent with a Rasmussen aneurysm (red arrow).

A computed tomography angiography (CTA) chest showed an irregular right upper lobe and right hilar 82 x 57 x 65 mm thick-walled complex cavitary structure containing fluid and gas favoring an infectious etiology such as TB (Figure 2).

**Figure 3:**
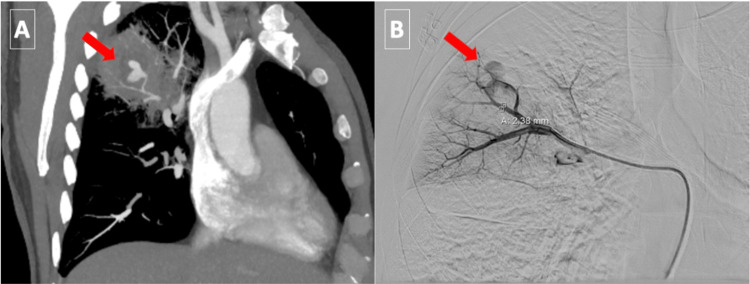
CTA chest A: CTA chest right pulmonary artery oblique maximum intensity projection (MIP) view demonstrating hyperdense focus of contrast extravasation and Rasmussen aneurysm (red arrow). B: Angiography of the segmental pulmonary artery to the right upper lobe demonstrating a Rasmussen aneurysm (red arrow). CTA: computed tomography angiography.

There was a hyperdense focus of contrast extravasation within the lesion measuring 17 x 12 x 13 mm with a feeding pulmonary arterial branch demonstrating a pseudoaneurysm and hemorrhage, consistent with a Rasmussen aneurysm (Figure 3A). The patient was started on piperacillin-tazobactam due to concerns about an infected pulmonary cavity and was admitted to the medical intensive care unit for close observation. The patient continued to have mild to moderate hemoptysis, with a dropping hemoglobin level to 9.4 G/DL. On hospital admission day 2, interventional radiology performed a pulmonary angiography demonstrating a Rasmussen aneurysm of a segmental pulmonary artery to the right upper lobe (Figure 3B).

**Figure 4:**
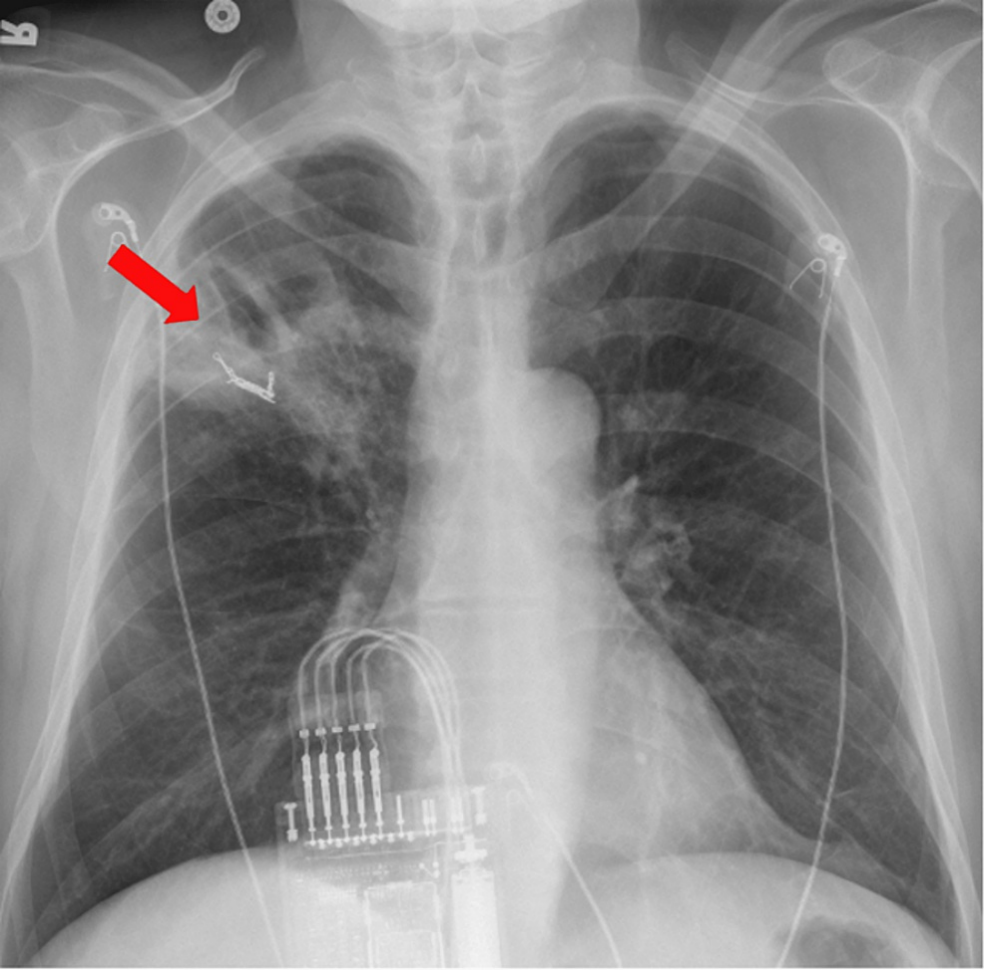
Repeat chest radiograph on hospital admission day 6 shows interval decrease of the right upper lobe cavitary lesion as well as coils from the embolization (red arrow).

Coil embolization of the tertiary feeding arteries was performed with excellent angiographic results. The patient continued to improve clinically, reporting minimal cough and no further hemoptysis. His leukocytosis remained stable, and blood cultures were negative. A repeat chest radiograph on hospital admission day 6 demonstrated an interval decrease in the size of the right upper lobe cavitary lesion (Figure 4).

Serology results for HIV, *Aspergillus*, *Histoplasma,* and *Coccidioides* returned negative. Echocardiography showed no myocardial, valvular, or pericardial disease. Three samples of expectorated sputum were tested for TB using smear fluorescence microscopy, direct nucleic acid amplification for *Mycobacterium tuberculosis,* and culture. All results were negative, no acid-fast bacilli were isolated after six weeks. The patient was discharged on hospital admission day 7 with amoxicillin/clavulanic acid and planned outpatient re-evaluation.

## Discussion

There are many causes of hemoptysis: autoimmune disease, hematologic disease, cardiac disease, airway disease, bronchial neoplasms, pulmonary parenchymal diseases, pulmonary vascular disorders, trauma or iatrogenic association, and infections such as TB or aspergillosis [1]. Causes of hemoptysis vary depending upon the geographical location in which a patient presents [7]. A Rasmussen aneurysm, typically presenting with hemoptysis, has originally been described as associated with pulmonary TB, with a higher prevalence seen in endemic regions, but remains a rare, sometimes forgotten, complication. TB sequelae structural complications include bronchiectasis, lung fibrosis, cavitation, and Rasmussen aneurysm to name a few [8]. Our case did not show evidence of active TB, although it remains likely that this patient, notably from an endemic region, had prior TB disease leading to the right upper lobe cavitary lesion demonstrated on the chest radiograph. Of note, few cases have reported non-TB cavitary structures in association with a Rasmussen aneurysm; however, these cases usually report other infectious etiologies, such as septic pulmonary embolic disease with findings of cavitary pulmonary nodules [7]. Though reported, little is published about the occurrence of sputum culture-negative pulmonary TB [9,10].

Our case identified the pulmonary pseudoaneurysm in association with a cavitary lesion. As the pulmonary artery branch encroaches upon the outer wall of the cavity, there is a progressive extension of the granulation tissue into the blood vessel wall, resulting in elastic fiber destruction and development of an aneurysm [11]. A pseudoaneurysm, not contained by all three layers of the vascular wall, is unstable and can easily bleed into the lung parenchyma or bronchial tree [7]. A Rasmussen aneurysm is an inflammatory pseudoaneurysmal dilatation of a pulmonary artery branch arising in segmental pulmonary arteries secondary to the gradual weakening of the adjacent pulmonary arterial wall [4,12,13]. CT pulmonary angiography is the imaging modality of choice to confirm this disease in patients with hemoptysis [14]. Endovascular occlusion using gel foam, arterial coiling, or stent grafts are the treatment modalities of choice, with urgent angiographic endovascular embolization being preferred due to its low invasiveness [13,14]. Surgical resection of the affected pulmonary lobe is reserved for refractory bleeding [3]. Pulmonary artery pseudoaneurysms cause the minority of cases of hemoptysis, and although they may have catastrophic outcomes, they are frequently missed on CT [7]. Therefore, diagnostic radiology should be familiar with the CT findings of pulmonary artery pseudoaneurysm to aid in appropriate recognition and treatment planning [7].

Because of the significant role and importance of diagnostic radiology in this case, aiding in timely identification and intervention, it is beneficial to discuss other etiologies of hemoptysis and their findings on imaging. It is necessary to differentiate a Rasmussen aneurysm from a bronchial artery pseudoaneurysm due to different interventional routes of approach for treatment; embolization through the pulmonary artery versus selective cannulization of the bronchial artery, respectively [5]. A bronchial artery pseudoaneurysm is a very rare, life-threatening disease observed in up to 3.9% of all cases of selective bronchial arteriography in patients with reported hemoptysis [15,16]. Its etiology remains unknown, but it is frequently noted in association with bronchiectasis, pulmonary aspergillosis, and vascular anomalies such as Rendu-Osler-Weber and Behcet diseases [15,16]. Vessel dilatation, tortuosity, and hypervascularity in association with aneurysm formation are findings seen on CTA [15]. Additionally, various pulmonary vascular abnormalities can lead to hemoptysis, and understanding potential underlying mechanisms as well as their features on imaging is required for accurate identification and interpretation. Bronchial-pulmonary and arteriovenous (AV) communications are disposed to rupture through pseudoaneurysm formation [13]. On CT, AV malformations are typically identified by recognizing the enlarged, tortuous feeding artery and enlarged draining vein [5]. Imaging for our case identified an adjacent cavitary lesion and absence of a nidus or draining vein, further demonstrating a Rasmussen aneurysm.

## Conclusions

This case demonstrates a rare pulmonary artery pseudoaneurysm, simply known as Rasmussen aneurysm, that was successfully managed with coil embolization of the tertiary feeding arteries. It emphasizes the need for understanding the numerous causes of hemoptysis with various etiologies and remembering the specific associated radiographic findings on CT for timely identification. This case highlights the importance of considering the disease for patients with hemoptysis and adds to the literature for performing embolization of the feeding vessels as a tool for definitive therapy.
